# Prevalence of *Helicobacter pylori* in Non-Cardia Gastric Cancer in China: A Systematic Review and Meta-Analysis

**DOI:** 10.3389/fonc.2022.850389

**Published:** 2022-05-03

**Authors:** Yong Lu, Fei Xiao, Yuan Wang, Zhenyu Wang, Di Liu, Feng Hong

**Affiliations:** ^1^School of Public Health, the Key Laboratory of Environmental Pollution Monitoring and Disease Control, Ministry of Education, Guizhou Medical University, Guiyang, China; ^2^State Key Laboratory of Ophthalmology, Zhongshan Ophthalmic Center, Sun Yat-sen University, Guangzhou, China; ^3^School of Public Health, Guangzhou Medical University, Guangzhou, China

**Keywords:** prevalence, *Helicobacter pylori*, non-cardia gastric cancer, meta-analysis, China

## Abstract

Non-cardia gastric cancer was significantly associated with *Helicobacter pylori* (*H. pylori*) infection. Reducing *H. pylori* prevalence was an important prevention strategy for non-cardia gastric cancer. However, national-level data on the *H. pylori* prevalence in non-cardia gastric cancer were limited in China. Therefore, we conducted this study to estimate the pooled prevalence of *H. pylori* in non-cardia gastric cancer in China. We searched PubMed, Embase, the Cochrane Library, China National Knowledge Infrastructure (CNKI), Wan Fang, and VIP Database for Chinese Technical Periodicals for studies reporting *H. pylori* prevalence in non-cardia gastric cancer in China which were published before September 1, 2021. Pooled prevalence was calculated using a random-effect model. Subgroup analysis and meta-regression were used to explore the potential sources of heterogeneity. Egger’s test and funnel plot were used to assess publication bias. A total number of 55 studies with 5324 cases of non-cardia gastric cancer were included in this study. The pooled prevalence of *H. pylori* in non-cardia gastric cancer in China was 66.5% (95%CI: 62%-71%, *I*^2^=93.8%, *P*<0.0001). In subgroup analysis, a significant difference in the prevalence of *H. pylori* in non-cardia gastric cancer was noted when stratified by geographic region of China (*P*=0.0112). The highest *H. pylori* prevalence (78.9%, 95%CI: 69.9%-87.8%) was noted in Northwest China and the lowest (53.1%, 95%CI: 38.9%-67.3%) was in North China. In meta-regression, a significant association between *H. pylori* prevalence and geographic region was found, while type of sample, *H. pylori* testing method, diagnosis period, detection timing, type of study design, quality grade, publication year, and sample size were not associated with the prevalence of *H. pylori* in non-cardia gastric cancer (*P*>0.05). A large proportion of non-cardia gastric cancers were associated with *H. pylori* infection in China, emphasizing the possible benefits of *H. pylori* eradication for the prevention and control of non-cardia gastric cancer.

## Introduction

Gastric cancer is one of the most common malignant tumors. In 2020, it was estimated that there were 1.09 million new gastric cancer cases and 0.77 million deaths from gastric cancer all over the world. Among all the new gastric cancer cases, more than 40% occurred in China. Meanwhile, gastric cancer caused about 12.4% of all cancer-related deaths, making it the third leading cause of cancer-related deaths in China (1).

Gastric cancer can be classified into two categories according to anatomical subsites: cardia gastric cancer and non-cardia gastric cancer (2–4). Due to different epidemiological characteristics and distinct pathogeneses, cardia and non-cardia gastric cancer are treated as two different diseases. Non-cardia gastric cancer is more common than cardia gastric cancer. In 2018, non-cardia gastric cancer accounted for up to 82% (0.85/1.03 million) of all gastric cancer cases around the world (2, 5). Regarding pathogeneses, *Helicobacter pylori* (*H. pylori*) has been proven to be one of the most important risk factors for non-cardia gastric cancer, with approximately 90% of non-cardia gastric cancer cases attributable to *H. pylori* infection worldwide in 2018 (5, 6), whereas no association was found between cardia gastric cancer and *H. pylori* infection (7, 8).

Given the strong association between non-cardia gastric cancer and *H. pylori* infection, reducing *H. pylori* prevalence has been listed as an important primary prevention strategy for gastric cancer prevention (9–11). Reliable estimation of *H. pylori* prevalence in non-cardia gastric cancer may be essential to the control and prevention of non-cardia gastric cancer, policy-making, and health resource allocation. To date, lots of studies conducted in China have reported the prevalence of *H. pylori* in non-cardia gastric cancer. However, the prevalence varied greatly across studies (12, 13). To the best of our knowledge, there is no study pooling the prevalence of *H. pylori* in non-cardia gastric cancer at the national level. Therefore, the primary objective of this meta-analysis was to estimate the pooled prevalence of *H. pylori* in non-cardia gastric cancer in China. Additionally, we also explored potential causes of heterogeneity in the reported prevalence.

## Materials and Methods

### Data Sources and Searches Strategy

We conducted a systematic literature review and meta-analysis according to the Preferred Reporting Items for Systematic Reviews and Meta-Analyses (PRISMA) guidelines to identify Chinese and English language studies published before September 1, 2021, which examined the prevalence of *H. pylori* in non-cardia gastric cancer in China. Two investigators (XF and WY) independently searched the literature in the following English databases: PubMed, Embase, the Cochrane Library, and the following Chinese databases: China National Knowledge Infrastructure (CNKI), Wan Fang, and VIP Database for Chinese Technical Periodicals. The search terms included (“cardia”), and (“gastric” or “stomach”), and (“cancer” or “neoplasms”), and (“Helicobacter” or “pylori”), and (“China” or “Chinese”). Authors (XF and WY) independently reviewed the studies to identify eligible studies. The three authors (LY, XF, and WY) discussed inconsistencies to reach consensus. We also reviewed the reference lists of included articles to identify additional eligible studies.

### Eligibility Criteria

We set the inclusion criteria as follows (1): studies reporting the prevalence of *H. pylori* from at least 10 cases of non-cardia gastric cancer, regardless of study design (2). Study site(s) located in China and Chinese participants were required (3). Studies were original studies published in English or Chinese language in any journal (4). *H. pylori* testing methods were clearly mentioned. We excluded studies which involved populations with special characteristics (e.g., recurrent cases) and studies with purposively selected cases (e.g., only advanced stage cases or metastatic cases). If several articles were based on the same research population, the one with the largest sample size was kept.

### Data Extraction and Quality Assessment

Two authors (XF and WY) extracted the data independently, and the inconsistencies between the two authors were managed through discussion with third author (LY). We made a standardized data extraction sheet in Microsoft Excel to extract the following variables: title, first author, journal of publication, publication year, geographic location of study, study period, sex distribution, age of diagnosis (mean/median/range), type of sample, *H. pylori* testing method, detection timing, type of study design, sample size, the number of *H. pylori*-positive cases, and prevalence of *H. pylori* in non-cardia gastric cancer. When the required information could not be extracted directly from the article, we contacted the authors for relevant information at least two times.

The 11-item Cross-Sectional/Prevalence Study Quality Assessment Forms recommended by the Agency for Healthcare Research and Quality (AHRQ) were used to assess the methodological quality of included cross-sectional studies (14) and the Newcastle-Ottawa Scale (NOS) was used to assess the methodological quality of included case-control and cohort studies (15). The studies would then be classified as low quality, moderate quality, and high quality, if they had scores of 0-3, 4-7, and 8-11 for cross-sectional studies, and 0-3, 4-6, and 7-9 for case-control and cohort studies.

### Data Analysis

Cochran’s *Q* test and *I*^2^ index were used to identify the heterogeneity across study. As the results showed significant heterogeneity, random-effect model was hence used to calculate the pooled prevalence of *H. pylori* in non-cardia gastric cancer and 95% confidence intervals (CI), weighted by DerSimonian-Laird model (16, 17).

We conducted subgroup analysis and meta-regression to explore the potential sources of heterogeneity (18). Subgroup analyses were carried out by the geographic region of China (Northwest, Northeast, Southwest, South Central, East, North, and not specified [NS]) (19), province, type of sample (breath, tissue, blood, and other), *H. pylori* testing method (14C urea breath test, immunohistochemical staining, Giemsa stain, Polymerase Chain Reaction [PCR], rapid urease test, Enzyme linked immunosorbent assay [ELISA], and other), diagnosis period (before 1999, 2000-2004, 2005-2009, 2010-2014, 2015-2019, and other), detection timing (before treatment and NS), type of study design (cross-sectional study, case-control study, and cohort study), and quality grade. Univariate meta-regression was performed based on the following variables: geographic region of China, type of sample, *H. pylori* testing method, diagnosis period, detection timing, type of study design, quality grade, publication year, and sample size.

We used Egger’s test and funnel plot to assess publication bias (20). Sensitivity analysis was conducted using leave-one-out method, which omitted one study at a time and re-conducted statistical analysis, in order to evaluate the influence of each omitted study on the pooled prevalence. All analyses were conducted using package “meta” in R 4.1.1, and statistical significance level was set as 0.05 for two-sided tests.

## Results

### Studies Selection 

**Figure 1:**
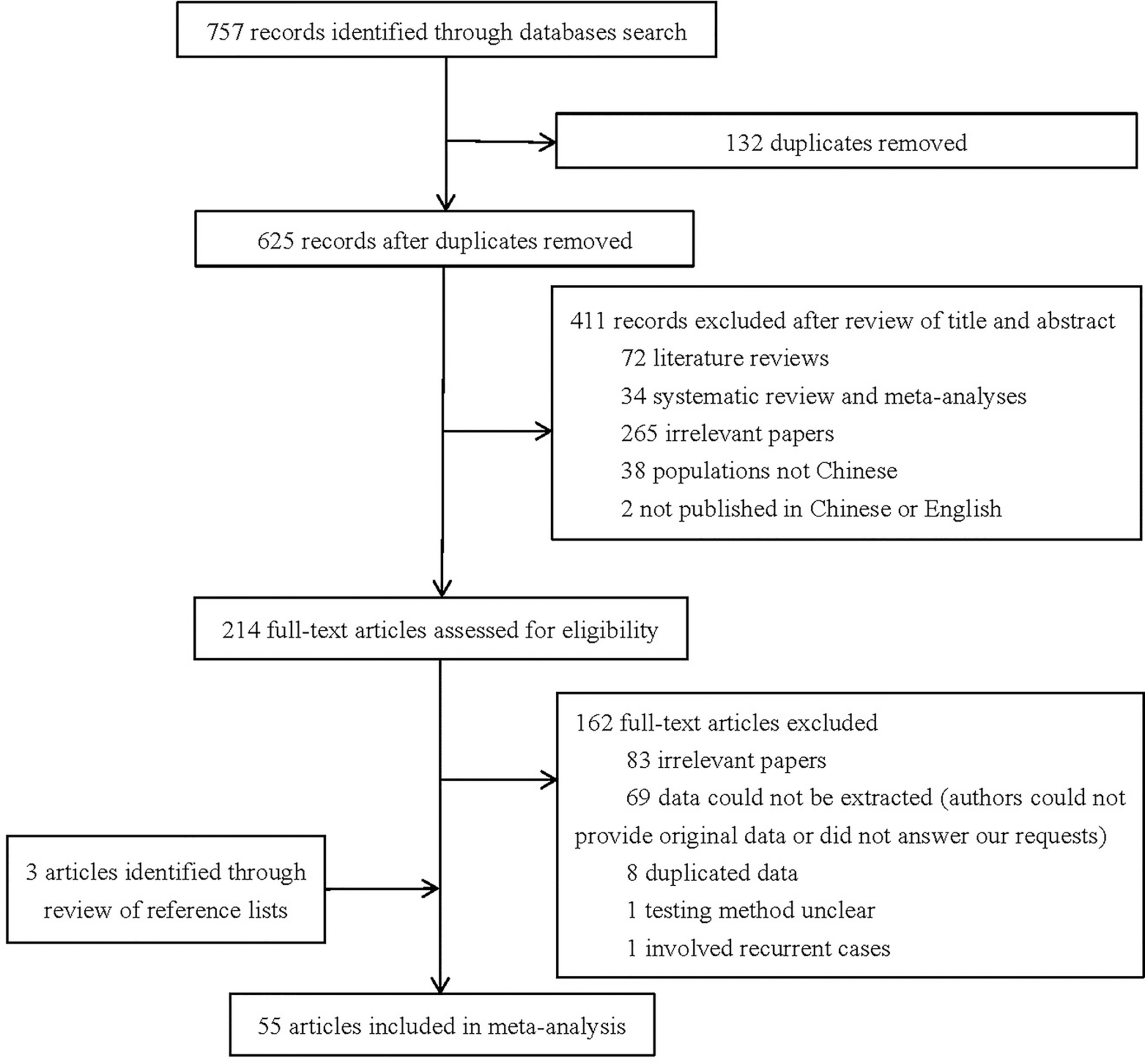
Flow diagram of studies selection.

A total number of 757 studies were initially identified through literature search. We excluded 132 studies because of duplication. After examination of titles and abstracts, 411 studies were excluded as they did not meet the eligibility criteria. After full-text review, 162 studies were excluded for the following reasons (1): 83 studies were not relevant to our study (2); 69 studies had no available data (authors could not provide original data or did not answer our requests) (3); eight studies reused data included in other studies (4); one study did not mention testing method of *H. pylori* (5); one study involved recurrent cases. Through checking reference lists, three additional relevant studies were added. Ultimately, 55 studies with 5324 cases of non-cardia gastric cancer were enrolled in the final meta-analysis. The detailed process of studies selection is shown in **Figure 1**.

### Characteristics of Included Studies

The detailed characteristics of the 55 included studies are summarized in **Supplementary Table S1**. The included studies were published between 1996 and 2020, and the sample size ranged from 10 to 343. Moreover, the majority of the studies were published in Chinese (44/55, 80%), and others were published in English. Of all the 55 studies, 17 were conducted in East China, 16 in South Central China, 10 in Northwest China, six in North China, three in Northeast China, one in Southwest China, and the geographic region of three studies was not specified. Regarding the type of sample, tissue was tested in 27 studies, blood in 18 studies, and breath in four studies. The most common *H. pylori* testing method was ELISA (n=15), followed by immunohistochemical staining (n=6), PCR (n=4), 14C urea breath test (n=4), Giemsa stain (n=3), and rapid urease test (n=3). Regarding type of study design, more than half (34/55, 61.8%) of included studies were cross-sectional studies. Additionally, about half of the studies (n=27) were rated as moderate quality, and 18 studies were regarded as high quality and 10 as low quality.

### Prevalence of *H. pylori*

**Figure 2:**
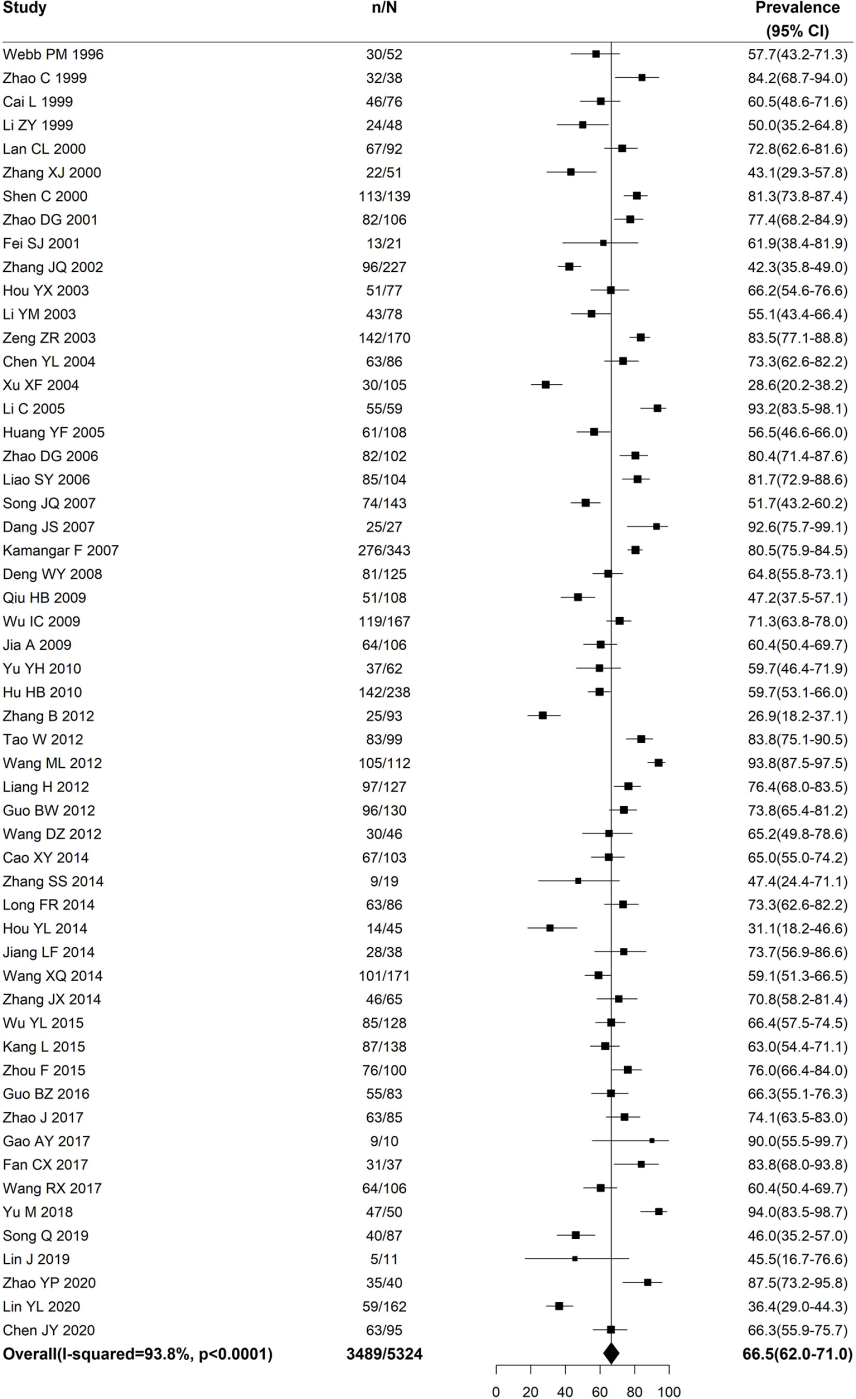
Forest plot for the pooled prevalence of *Helicobacter pylori* in non-cardia gastric cancer in China.

**Figure 2** shows that the pooled prevalence of *H. pylori* in non-cardia gastric cancer in China was 66.5% (95%CI: 62%-71%). Statistical heterogeneity was observed among the included studies (*I*^2^=93.8%, *P*<0.0001).

### Subgroup Analysis

**Table 1** shows the results of subgroup analysis. Significant differences in the prevalence of *H. pylori* in non-cardia gastric cancer was noted when stratified by geographic region of China (*P*=0.0112) and type of sample (*P*=0.0326). The geographic region with the highest prevalence of *H. pylori* was Northwest China (78.9%, 95%CI: 69.9%-87.8%) followed by Northeast China (74.3%, 95%CI: 61.9%-86.7%), and Southwest China (72.8%, 95%CI: 63.7%-81.9%), while the geographic region with the lowest prevalence was North China (53.1%, 95%CI: 38.9%-67.3%). Additionally, when stratified by province, the prevalence of *H. pylori* in non-cardia gastric cancer was also statistically different (*P*<0.0001). The top three provinces with the highest prevalence of *H. pylori* were Heilongjiang (90%, 95%CI: 71.4%-100%), Ningxia (89.2%, 95%CI: 79.5%-98.9%), and Shandong (84.2%, 95%CI: 72.6%-95.8%), whereas Beijing (43.1%, 95%CI: 29.5%-56.7%), Inner Mongolia (45.4%, 95%CI: 24.7%-66%), and Fujian (49.2%, 95%CI: 35.2%-63.3%) showed the lowest prevalence of *H. pylori*. The prevalence of *H. pylori* detected in breath (78.7%, 95%CI: 67.2%-90.2%) was significantly higher than that detected in tissue (67%, 95%CI: 60.7%-73.4%) and blood (64.1%, 95%CI: 54.8%-73.4%). The prevalence of *H. pylori* in non-cardia gastric cancer was generally similar for different *H. pylori* testing methods (*P*=0.4846), diagnosis periods (*P*=0.2623), detection timings (*P*=0.4719), types of study design, (*P*=0.6952), and quality grades (*P*=0.7712).

**Table 1:** Prevalence of *Helicobacter pylori* in non-cardia gastric cancer by stratification variables.

	Studies	N	*H. pylori* positive cases	Pooled *H. pylori* prevalence(95% CI)	*P* value	Heterogeneity	
						*I*^2^(%)	*P* value
**Geographic region**					0.0112		
Northwest China	10	880	657	78.9 (69.9; 87.8)		92.9	<0.0001
Northeast China	3	198	139	74.3 (61.9; 86.7)		66.6	0.0502
Southwest China	1	92	67	72.8 (63.7; 81.9)		–	–
South Central China	16	1866	1307	69.9 (63.5; 76.4)		90.2	<0.0001
East China	17	1543	911	62.2 (53.8; 70.6)		93.4	<0.0001
North China	6	545	300	53.1 (38.9; 67.3)		93.2	<0.0001
NS	3	200	108	51.2 (31.2; 71.2)		87.6	0.0003
**Province**					<0.0001		
Heilongjiang	1	10	9	90.0 (71.4; 100.0)		–	–
Ningxia	2	211	188	89.2 (79.5; 98.9)		80.7	0.0227
Shandong	1	38	32	84.2 (72.6; 95.8)		–	–
Gansu	2	123	94	77.9 (64.8; 83.4)		46.3	0.1723
Shaanxi	6	546	375	75.5 (61.7; 89.2)		94.3	<0.0001
Henan	7	954	723	74.9 (70.3; 79.5)		60.6	0.0186
Liaoning	1	85	63	74.1 (64.8; 83.4)		–	–
Chongqing	1	92	67	72.8 (63.7; 81.9)		–	–
Hebei	2	176	126	71.5 (64.0; 79.0)		13.8	0.2814
Taiwan	1	167	119	71.3 (64.4; 78.1)		–	–
Jiangsu	7	397	294	70.7 (59.8; 81.6)		77.2	0.0002
Guangdong	5	453	304	67.5 (54.5; 80.4)		90.5	<0.0001
Hubei	3	221	138	65.1 (35.8; 94.4)		97.0	<0.0001
Jilin	1	103	67	65.1 (55.8; 74.3)		–	–
Guangxi	1	238	142	59.7 (53.4; 65.9)		–	–
Shanghai	3	365	189	57.5 (39.3; 75.7)		93.1	<0.0001
Fujian	5	576	277	49.2 (35.2; 63.3)		92.4	<0.0001
Inner Mongolia	3	318	152	45.4 (24.7; 66.0)		94.2	<0.0001
Beijing	1	51	22	43.1 (29.5; 56.7)		–	–
NS	3	200	108	51.2 (31.2; 71.2)		87.6	0.0003
**Type of sample**					0.0326		
Breath	4	278	204	78.7 (67.2; 90.2)		83.2	0.0005
Tissue	27	2452	1577	67.0 (60.7; 73.4)		92.7	<0.0001
Blood	18	2036	1367	64.1 (54.8; 73.4)		96.2	<0.0001
Other^a^	6	558	341	61.4 (57.3; 65.4)		0.0	0.5004
***H. pylori* testing method**					0.4846		
14C urea breath test	4	278	204	78.7 (67.2; 90.2)		83.2	0.0005
Immunohistochemical Staining	6	355	250	71.1 (63.9; 78.3)		56.9	0.0408
Giemsa stain	3	412	242	66.4 (41.8; 91.0)		97.4	<0.0001
PCR method	4	514	321	66.0 (50.5; 81.5)		90.7	<0.0001
Rapid urease test	3	337	187	65.1 (32.8; 97.8)		98.5	<0.0001
ELISA	15	1746	1138	62.4 (52.4; 72.4)		96.0	<0.0001
Other^b^	20	1682	1147	66.4 (59.8; 73.1)		91.0	<0.0001
**Diagnosis period**					0.2623		
Before1999	6	488	338	64.7 (52.2; 77.2)		87.7	<0.0001
2000-2004	3	299	154	52.7 (27.1; 78.3)		95.9	<0.0001
2005-2009	3	390	236	60.6 (55.7; 65.4)		0.0	0.7708
2010-2014	7	591	410	69.5 (62.5; 76.5)		73.3	0.0010
2015-2019	4	188	127	69.8 (44.1; 95.5)		95.4	<0.0001
Other^c^	32	3368	2224	67.6 (61.4; 73.8)		94.8	<0.0001
**Detection timing**					0.4719		
Before treatment	19	1960	1192	64.1 (56.1; 72.2)		93.9	<0.0001
NS	36	3364	2297	67.7 (62.2; 73.1)		93.3	<0.0001
**Type of study design**					0.6952		
Cross-sectional study	34	2698	1784	67.9 (62.3; 73.4)		92.2	<0.0001
Case-control study	19	2231	1399	63.8 (55.5; 72.1)		95.4	<0.0001
Cohort study	2	395	306	70.0 (47.8; 92.3)		90.1	0.0015
**Quality grade**					0.7712		
High	18	2278	1480	64.1 (55.5; 72.8)		95.8	<0.0001
Moderate	27	2222	1420	67.2 (61.2; 73.2)		91.0	<0.0001
Low	10	824	589	68.9 (58.0; 79.7)		93.3	<0.0001

H. pylori, Helicobacter pylori; CI, confidence interval; NS, not specific; PCR, polymerase chain reaction; ELISA, enzyme linked immunosorbent assay.

a using several types of samples.

b using other testing method or several testing methods.

c The diagnosis period could not be divided into corresponding group or the diagnosis period was not clear.

### Meta-Regression

The results of univariate meta-regression indicated that there was significant association between geographic region of China and the prevalence of *H. pylori* in non-cardia gastric cancer (**Table 2**). Compared with the prevalence in North China, the prevalence was higher in Northwest China (*β*=0.2570, 95%CI: 0.1002-0.4138, *P*=0.0013), Northeast China (*β*=0.2216, 95%CI: 0.0016-0.4417, *P*=0.0484), and South Central China (*β*=0.1663, 95%CI: 0.0202-0.3124, *P*=0.0257). The results of univariate meta-regression also revealed that type of sample, *H. pylori* testing method, diagnosis period, detection timing, type of study design, quality grade, publication year, and sample size were not significantly associated with the prevalence of *H. pylori* in non-cardia gastric cancer (*P*>0.05).

**Table 2:** Assessing the effect of study variables on the pooled prevalence of *Helicobacter pylori* in non-cardia gastric cancer in China using univariable meta-regression analysis.

Variable	*β* (95% CI)	*SE*	*P* value
**Geographic region**			
Northwest China	0.2570 (0.1002; 0.4138)	0.0800	0.0013
Southwest China	0.1969 (-0.1293; 0.5232)	0.1665	0.2368
Northeast China	0.2216 (0.0016; 0.4417)	0.1123	0.0484
South Central China	0.1663 (0.0202; 0.3124)	0.0745	0.0257
East China	0.0907 (-0.0552; 0.2366)	0.0744	0.2233
NS	-0.0185 (-0.2370; 0.2000)	0.1115	0.8684
North China	Reference		
**Type of sample**			
Breath	0.1674 (-0.0479; 0.3826)	0.1098	0.1275
Tissue	0.0488 (-0.1023; 0.1999)	0.0771	0.5265
Blood	0.0218 (-0.1358; 0.1794)	0.0804	0.7862
Other^a^	Reference		
***H. pylori* testing method**			
14C urea breath test	0.1268 (-0.0607; 0.3143)	0.0957	0.1851
Immunohistochemical Staining	0.0415 (-0.1231; 0.2061)	0.0840	0.6213
Rapid urease test	-0.0101 (-0.2187; 0.1984)	0.1064	0.9240
Giemsa stain	0.0012 (-0.2077; 0.2101)	0.1066	0.9910
PCR method	-0.0014 (-0.1877; 0.1848)	0.0950	0.9879
ELISA	-0.0374 (-0.1544; 0.0797)	0.0597	0.5316
Other^b^	Reference		
**Diagnosis period**			
Other^c^	-0.0355 (-0.2223; 0.1514)	0.0954	0.7100
Before1999	-0.0667 (-0.2920; 0.1586)	0.1150	0.5618
2000-2004	-0.1847 (-0.4471; 0.0777)	0.1339	0.1678
2005-2009	-0.0952 (-0.3588; 0.1684)	0.1345	0.4789
2010-2014	-0.0265 (-0.2461; 0.1931)	0.1120	0.8131
2015-2019	Reference		
**Detection timing**			
Before treatment	-0.0352 (-0.1301; 0.0598)	0.0484	0.4678
NS	Reference		
**Type of study design**			
Cross-sectional study	-0.0210 (-0.2646; 0.2226)	0.1243	0.8657
Case-control study	-0.0609 (-0.3093; 0.1875)	0.1267	0.6308
Cohort study	Reference		
**Quality grade**			
High	-0.0471 (-0.1797; 0.0855)	0.0677	0.4865
Moderate	-0.0173 (-0.1430; 0.1085)	0.0642	0.7875
Low	Reference		
**Publication year**	0.0008 (-0.0068; 0.0078)	0.0036	0.8243
**Sample size**	-0.0002 (-0.0010; 0.0006)	0.0004	0.6018

SE, standard error; CI, confidence interval; NS, not specific; PCR, polymerase chain reaction; ELISA, enzyme linked immunosorbent assay; H. pylori, Helicobacter pylori.

a using several types of samples.

b using other testing method or several testing methods.

c The diagnosis period could not be divided into corresponding group or the diagnosis period was not clear.

### Publication Bias and Sensitivity Analysis

**Figure 3:**
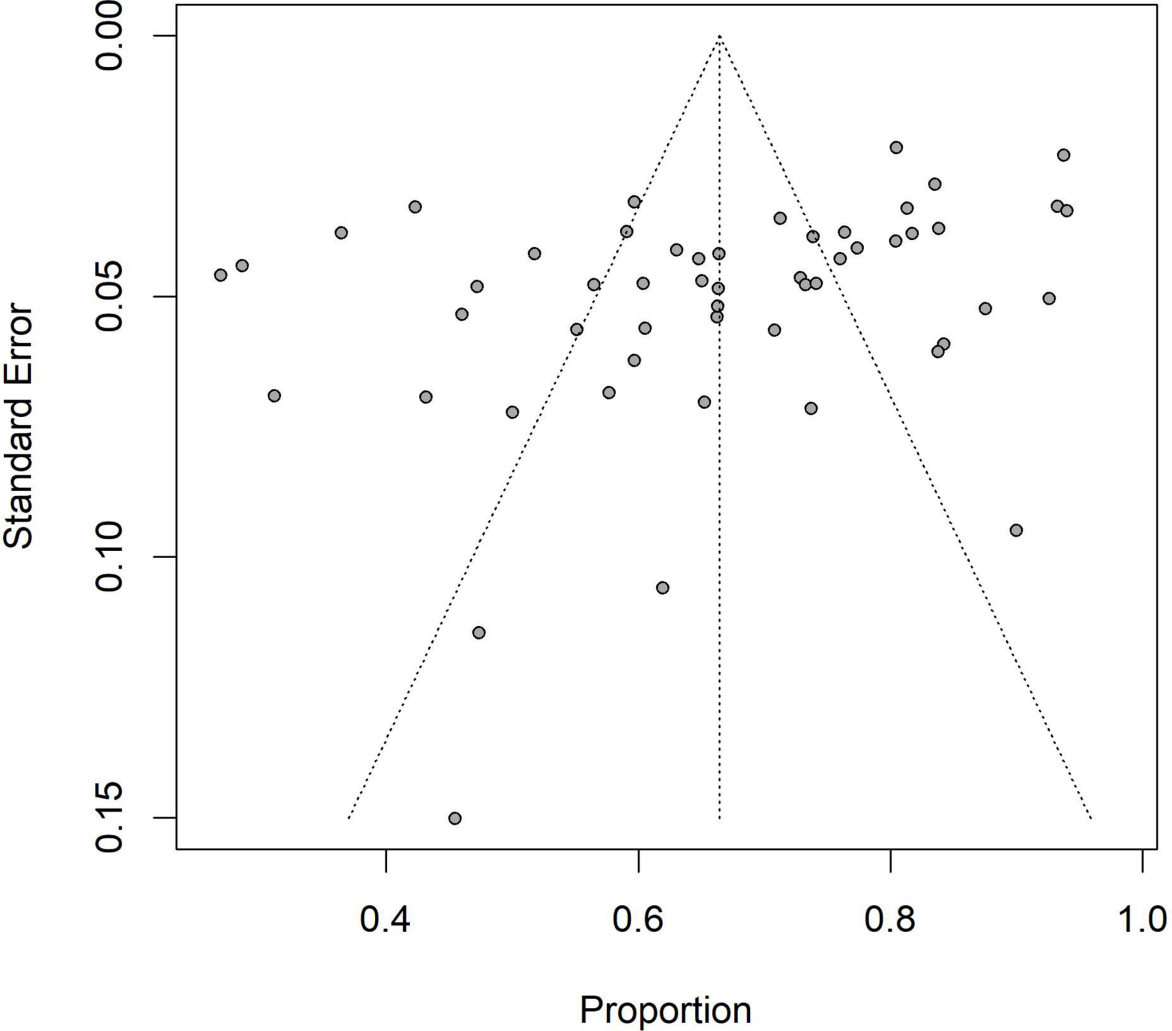
Funnel plot for the pooled prevalence of *Helicobacter pylori* in non-cardia gastric cancer in China.

The Egger’s test for funnel plot (**Figure 3**) asymmetry was significant (*t*=-3.01, *P*=0.0040), indicating that there was obvious publication bias in all studies. The results of sensitivity analysis indicated that the lowest prevalence of *H. pylori* in non-cardia gastric cancer was 65.92% (95%CI: 0.6146-0.7038) when the Wang ML et al. study was omitted, and the highest prevalence was 67.25% (95%CI: 0.6292-0.7157) when the Zhang B study was omitted. The omission of studies did not significantly modify the pooled prevalence (**Supplementary Figure S1**).

## Discussion

Based on 5324 non-cardia gastric cancer patients from 55 studies, which covered more than half of provinces and autonomous regions in China, we conducted a meta-analysis to estimate the prevalence of *H. pylori* in non-cardia gastric cancer patients at the national level for the first time. We reported a comprehensive estimate of *H. pylori* prevalence in non-cardia gastric cancer patients in China at 66.5% (95%CI: 62%-71%). Moreover, substantial geographic variations were noted in the prevalence of *H. pylori*, which was the highest in Northwest China and the lowest in North China.

The results of our study indicated that the pooled prevalence of *H. pylori* in non-cardia gastric cancer was 66.5% (95%CI: 62%-71%) in China. The prevalence of *H. pylori* in non-cardia gastric cancer in different countries varied greatly, such as 38.5% in the United States, 57% in Spain, 79.5% in South Korea, and 91.8% in Japan (4, 21–23). This difference might be explained by the following reasons: socio-economic status, dietary habits, and racial disparities. Suerbaum S et al. have mentioned that there was a strong inverse correlation between *H. pylori* prevalence and socio-economic status. Populations with lower socio-economic status were more likely to be infected with *H. pylori* (24, 25). Lower social status was commonly accompanied by crowded living conditions and poor hygienic conditions, which might increase the risk of acquisition and transmission of *H. pylori* (24, 26). Several previous studies have revealed that the consumption of high-salt foods (such as pickles and preserved products) might be associated with an increased chance of *H. pylori* infection (27–29). Tsugane S et al. speculated that a high-salt diet might induce gastric mucosa damage and destroy mucosal barrier. These changes in gastric mucosa might lead to an increase in *H. pylori* infection (29). Data based on National Health and Nutrition Examination Surveys of the United States have also shown that racial disparities played a certain role in the prevalence of *H. pylori*. Compared with *H. pylori* prevalence in whites, the prevalence in African Americans was higher (30).

Several previous studies, especially in Japan, reported that the *H. pylori* prevalence in gastric cancer was as high as 99% when strict criteria were used to diagnose *H. pylori* infection (31–33). Some other studies in Japan and South Korea used multiple testing methods to diagnose *H. pylori* infection, which also showed that the *H. pylori* prevalence in gastric cancer patients was above 90% (34–36). The *H. pylori* prevalence of these studies was much higher than the pooled *H. pylori* prevalence in our study. There were several possible reasons for this difference. First, these previous studies used strict criteria or multiple testing methods to diagnose *H. pylori* infection, which could greatly increase the sensitivity of detection. Moreover, most participants of these studies were cases of gastric cancer, while the participants of our study were non-cardia gastric cancer. Finally, most of these studies were conducted in Japan or South Korea. The *H. pylori* prevalence varied in different countries and regions.

The results of our study indicated a significant heterogeneity in the prevalence of *H. pylori* in non-cardia gastric cancer across different geographical regions in China. The highest *H. pylori* prevalence was noted in Northwest China at 78.9%, which was consistent with the highest incidence of gastric cancer in this region reported by the 2016 Chinese Cancer Registry Annual Report (37). Meanwhile, in line with our findings showing that the lowest *H. pylori* prevalence in non-cardia gastric cancer was noted in North China, Zhang WD et al. have also mentioned that the lowest *H. pylori* prevalence among the general population was in North China (38). The socio-economic status might be the main factor accounting for this geographic region difference. Unbalanced socio-economic development in different regions would lead to differences in living environment, education level, hygiene conditions, as well as water source (26, 39–41). The combined effect of the above factors may have resulted in the variation of *H. pylori* prevalence in different geographic regions.

The prevalence of *H. pylori* in non-cardia gastric cancer also showed significant variations across different provinces, and the highest *H. pylori* prevalence was noted in Heilongjiang Province at 90%. However, the estimate of Heilongjiang Province was based on only 10 cases from one study, leading to a wide range of its 95% CI. Furthermore, there were another seven provinces where the estimates of *H. pylori* prevalence were also based on only one study with the sample size ranging from 38 to 238. At the provincial level, part of the estimates in our study were estimated in the basis of a relatively small sample size, which might result in unstable results. Therefore, the results should be interpreted with caution, and additional studies with a larger sample size are needed to confirm our findings in the future.

The prevalence of *H. pylori* in the general population and non-cardia gastric cancer cases from different geographic regions was not completely consistent. From January 2002 to June 2004, Zhang WD et al. conducted an epidemiological survey on the *H. pylori* prevalence among 26,341 general people from 19 provinces and autonomous regions in China. The study showed that the *H. pylori* prevalence in the general population from different geographic regions ranged from high to low as: 66.26% in Central China, 59.16% in Eastern China, 58.27% in Western China, 50.08% in Southern China, and 46.84% in Northern China. In terms of provinces, the *H. pylori* prevalence among general population in Tibet was the highest at 84.62%, while the *H. pylori* prevalence in Guangdong was the lowest at 42.01% (38). Our results indicated that the geographic region with the lowest *H. pylori* prevalence in non-cardia gastric cancer cases was noted in North China, which was in line with the previous survey conducted by Zhang WD et al. The possible reason for the difference in the distribution of *H. pylori* between general population and cases of non-cardia gastric cancer was that non-cardia gastric cancer was caused by a combination of various risk factors including *H. pylori* infection, dietary habits, ethnicity, smoking, radiation exposure, family history, etc. *H. pylori* infection is one of the most important factors, but not the only one (42).

Type of sample might have an influence on the estimates of prevalence of *H. pylori* in non-cardia gastric cancer. The results of subgroup analysis revealed significant heterogeneity across different types of samples, with the highest prevalence of *H. pylori* detected in breath (78.7%). However, the results of meta-regression indicated that the differences in *H. pylori* prevalence among different sample types were not significant. The disparity in results might be attributed to the small number of the studies included. This meta-analysis only included four studies with 278 cases that detected the *H. pylori* prevalence in breath. Spineli LM et al. have mentioned that in order to obtain robust results, at least 10 studies should be included for each covariate in meta-regression implementation. In order to further confirm our finding, more studies detecting the *H. pylori* prevalence in breath will be needed in the future (43). In line with our findings, Liao YQ et al. also mentioned that the prevalence of *H. pylori* detected in breath would be higher than that in other samples. This might be partly because *H. pylori* infection detected in breath could reflect the *H. pylori* infection status in the whole gastrointestinal tract. Other pathogenic microorganisms in the gastrointestinal tract that could produce urease might cause false positive in the detection, which might increase the positive rate of *H. pylori* (44, 45).

The results of subgroup analysis and meta-regression both showed that the prevalence of *H. pylori* in non-cardia gastric cancer was not significantly associated with different *H. pylori* testing methods. This finding was in agreement with a recent study estimating the prevalence of *H. pylori* in cases with gastrointestinal diseases other than gastric cancer, which showed that the *H. pylori* prevalence detected by different *H. pylori* testing methods were generally similar (46). In our study, all *H. pylori* detected in breath was tested using 14C urea breath test. However, type of sample was significantly associated with *H. pylori* prevalence, while the relationship between testing method and *H. pylori* prevalence was not significant. Spineli LM et al. have revealed that when the subgroups contained fewer studies, the subgroup analysis could be underpowered to test the relationship between variables (43). Borenstein M et al. also indicated that one of the key factors driving the precision of subgroup analysis was the number of studies (47). Compared with type of sample, *H. pylori* testing method contained more subgroups, resulting in less studies in each subgroup. This may be the possible reason that the results of the above two subgroup analyses were different.

The studies included in our meta-analysis used several single testing methods to detect *H. pylori* infection among non-cardia gastric cancer cases. However, several guidelines revealed that one single testing method could not be considered as the gold standard for *H. pylori* detection (48–50). These commonly used testing methods all have some disadvantages. For example, IgG would remain in the blood for months or years even after *H. pylori* was eradicated. As such, antibody-based tests (e.g., ELISA) could not distinguish between current and past infections (51, 52). Moreover, ELISA is less accurate than 14C urea breath test and the cut-off values need a local validation (48, 53). As for 14C urea breath test, several factors such as atrophy, bismuth, proton pump inhibitor (PPI), and antibiotics may lead to false-negative, and it also should be validated locally (48, 54). Several guidelines have recommended the combination of single testing methods (e.g., combination of a validated serology and urea breath test), which could improve the accuracy of the detection (48–50). However, in our meta-analysis, most included studies (43/55, 78.2%) used a single testing method to detect *H. pylori*, which might lead to bias in the pooled *H. pylori* prevalence among non-cardia gastric cancer cases in China. Because *H. pylori* infection tends to clear as non-cardia gastric cancer progresses and the detection of past *H. pylori* infection is difficult, especially in retrospective studies (19/55, 34.5%), the pooled *H. pylori* prevalence in Chinese non-cardia gastric cancer cases may be underestimated (35, 55, 56).

The results of our study also suggested that there was no correlation between the diagnosis period and the prevalence of *H. pylori* in non-cardia gastric cancer. Previous meta-analysis conducted by Qiao J et al. showed that the prevalence of *H. pylori* in cases with gastric cancer in China declined from 1996 to 2015 (57). However, no decreasing trend of *H. pylori* prevalence in non-cardia gastric cancer was observed in our study. This difference might be attributed to the different study population. The previous study included cases with gastric cancer, while the included cases in our study were limited as cases with non-cardia gastric cancer.

The “test and treat” strategy for *H. pylori* infection has been shown to be cost-effective in some western countries (e.g., the United Kingdom and the United States) (58, 59). Japan has included *H. pylori* eradication therapy into the coverage of national medical insurance, becoming the first country to implement universal *H. pylori* “test and treat” strategy worldwide (60). Even with high prevalence of *H. pylori* infection and gastric cancer, whether to implement the “test and treat” strategy for *H. pylori* infection among the general population in China was still controversial. Several factors such as cost-effectiveness, personal willingness, usage of antibiotic, gastric cancer incidence, and prevalence of *H. pylori* infection would affect the implementation of this strategy (59, 61). Our study pooled the *H. pylori* prevalence in non-cardia gastric cancer and explored the influence factors of pooled prevalence, which might have certain public health significance in providing evidence for the control and prevention of non-cardia gastric cancer, as well as for policy making and health resource allocation.

The strengths of this meta-analysis included the following items. To the best of our knowledge, this was the first meta-analysis that estimated the pooled prevalence of *H. pylori* in non-cardia gastric cancer in China. An overview of current studies about *H. pylori* prevalence in non-cardia gastric cancer conducted in China was presented in our study. Moreover, our study has included a large number of studies. Based on a large sample size of 5324 cases from 55 studies, we were able to obtain a relatively robust estimate of the pooled *H. pylori* prevalence in non-cardia gastric cancer.

There were several limitations in our meta-analysis. Even though subgroup analysis and meta-regression were performed to minimize the heterogeneity across the included studies, significant heterogeneity still could be observed in subgroup analysis. The factors included in our study could not well explain the heterogeneity, which might affect the generalizability of our results. Moreover, some important factors (e.g., dietary habit, drinking, and gender) could not be extracted from the included studies, which might have potential influence on the heterogeneity. Another limitation was that some estimates in our study (e.g., *H. pylori* prevalence in different provinces) were calculated based on the small number of cases. Therefore, these results should be interpreted with caution, and more studies are needed to further confirm these results in the future. Another limitation of our study was that single testing methods used in the included studies for detecting *H. pylori* infection all had some limitations, and the results could not be completely accurate, which may lead to a certain bias in the pooled *H. pylori* prevalence. Finally, significant publication bias may result in an underestimation of pooled *H. pylori* prevalence.

## Conclusions

This meta-analysis presented an overview of *H. pylori* prevalence in non-cardia gastric cancer in China. In conclusion, our study estimated the pooled prevalence of *H. pylori* in non-cardia gastric cancer was 66.5% (95%CI: 62%-71%) in China. Variation in *H. pylori* prevalence across different geographical regions was statistically significant, with the highest *H. pylori* prevalence (78.9%) in Northwest China and the lowest (53.1%) in North China. Type of sample might be associated with *H. pylori* prevalence, and further studies are needed to confirm this finding. A large proportion of non-cardia gastric cancers was associated with *H. pylori* infection, emphasizing the potential benefits of *H. pylori* eradication for reducing the disease burden of non-cardia gastric cancer. Our study might have certain public health significance in providing evidence for the control and prevention of non-cardia gastric cancer, as well as for policy making and health resource allocation.

## Data Availability Statement

The original contributions presented in the study are included in the article/**Supplementary Material**. Further inquiries can be directed to the corresponding author.

## Author Contributions

Study concepts: FH; Study design: FH; Data acquisition: FX and YW; Quality control of data and algorithms: YL and ZW; Data analysis and interpretation: YL and DL; Manuscript preparation: YL, FX, and YW; Manuscript editing: YL; Manuscript review: ZW, DL, and FH. All authors contributed to the article and approved the submitted version.

## Funding

This study was supported by the Doctoral Start-up Foundation of Guizhou Medical University [No. J (2020)65], National Natural Science Foundation of China Incubation Program, Guizhou Medical University [No. 20NSP061], the First-Class Discipline Construction Project in Guizhou Province Public Health and Preventive Medicine [No.2017 (85)], Foundation for the Establishment of Postdoctoral Mobile Station in Public Health and Preventive Medicine, Guizhou Medical University [41202020204], and Guizhou Basic Research (Science and Technology Fund) Project [ZK (2022) General 373]. All funding parties did not have any role in the design of the study or in the explanation of the data.

## Conflict of Interest

The authors declare that the research was conducted in the absence of any commercial or financial relationships that could be construed as a potential conflict of interest.

## Publisher’s Note

All claims expressed in this article are solely those of the authors and do not necessarily represent those of their affiliated organizations, or those of the publisher, the editors and the reviewers. Any product that may be evaluated in this article, or claim that may be made by its manufacturer, is not guaranteed or endorsed by the publisher.

## Abbreviations

*H. pylori*, *Helicobacter pylori*; CNKI, China National Knowledge Infrastructure; PRISMA, Preferred Reporting Items for Systematic Reviews and Meta-Analyses; AHRQ, Agency for Healthcare Research and Quality; NOS, Newcastle-Ottawa Scale; CI, confidence interval; PCR, Polymerase Chain Reaction; ELISA, Enzyme linked immunosorbent assay; SE, standard error; PPI, proton pump inhibitor.
