# Supplementary material for: Prevalence of Helicobacter pylori in Non-Cardia Gastric Cancer in China: A Systematic Review and Meta-Analysis
Source: Front Oncol. 2022 May 3;12:850389. doi: 10.3389/fonc.2022.850389 (PMC9111517; doi:10.3389/fonc.2022.850389)
Supplement: Supplementary file 1 [file DataSheet_1.docx]

# Supplementary Material

**Supplementary Figure S1:** Forest plot for sensitivity analysis

**Supplementary Table S1:** Characteristics of included studies about prevalence of *Helicobacter pylori* in non-cardia gastric cancer in China

**
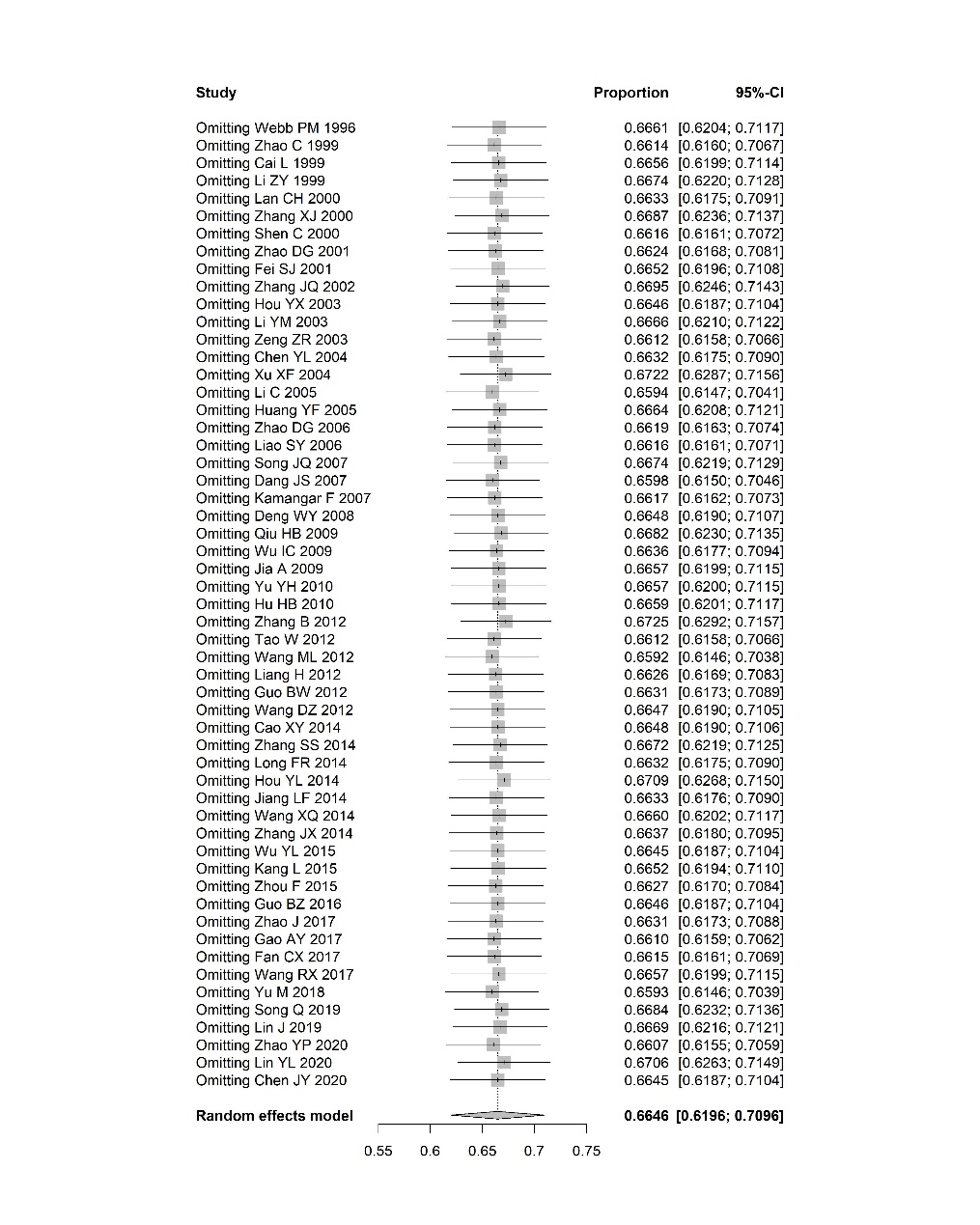
**

**Supplementary Figure S1.** Forest plot for sensitivity analysis

**Supplementary Table S1:** Characteristics of included studies about prevalence of *Helicobacter pylori* in non-cardia gastric cancer in China

| **Study** | **Province** | **District** | ***H. pylori* testing method** | **Type of**  **sample** | **Diagnosis period** | **Detection timing** | **Type of study design** | **Males**  **(%)** | **Quality**  **score** | **Grade** | **Sample**  **size** | **Positive**  **cases** |
| --- | --- | --- | --- | --- | --- | --- | --- | --- | --- | --- | --- | --- |
| Webb PM 1996 ([1](#_ENREF_1))[1] | Shanghai | East China | ELISA | Blood | 1986-1992 | NS | cohort study | NS | 6 | Moderate | 52 | 30 |
| Zhao C 1999 ([2](#_ENREF_2)) | Shandong | East China | PCR method | Tissue | NS | Before treatment | cross-sectional study | NS | 3 | Low | 38 | 32 |
| Cai L 1999 ([3](#_ENREF_3)) | Fujian | East China | ELISA | Blood | NS | NS | case-control study | NS | 7 | High | 76 | 46 |
| Li ZY 1999 ([4](#_ENREF_4)) | Jiangsu | East China | ELISA | Blood | 1997 | NS | cross-sectional study | NS | 5 | Moderate | 48 | 24 |
| Lan CH 2000 ([5](#_ENREF_5)) | Chongqing | Southwest China | Rapid urease test and Tissue staining | Tissue | 1998-1999 | NS | case-control study | NS | 8 | High | 92 | 67 |
| Zhang XJ 2000 ([6](#_ENREF_6)) | Beijing | North China | Silver staining and rapid urease test | Tissue | 1995-1998 | NS | cross-sectional study | 71.8 | 4 | Moderate | 51 | 22 |
| Shen C 2000 ([7](#_ENREF_7)) | Jiangsu | East China | Giemsa stain and CLO test | Tissue | 1994-1999 | NS | cross-sectional study | NS | 3 | Low | 139 | 113 |
| Zhao DG 2001 ([8](#_ENREF_8)) | Henan | South Central China | Urease test and tissue staining | Tissue | 1995-1999 | NS | cross-sectional study | NS | 3 | Low | 106 | 82 |
| Fei SJ 2001 ([9](#_ENREF_9)) | Jiangsu | East China | Toluidine blue staining | Tissue | NS | NS | cross-sectional study | NS | 1 | Low | 21 | 13 |
| Zhang JQ 2002 ([10](#_ENREF_10)) | Shanghai | East China | Giemsa stain | Tissue | 1998-2002 | Before treatment | cross-sectional study | 65.5 | 4 | Moderate | 227 | 96 |
| Hou YX 2003 ([11](#_ENREF_11)) | NS | NS | Ag-NOR argentophilic staining method | Tissue | NS | Before treatment | cross-sectional study | 68.9 | 1 | Low | 77 | 51 |
| Li YM 2003 ([12](#_ENREF_12)) | NS | NS | Triple staining | Tissue | NS | NS | cross-sectional study | NS | 2 | Low | 78 | 43 |
| Zeng ZR 2003 ([13](#_ENREF_13)) | Guangdong&Shaanxi | South Central & Northwest China | ELISA | Blood | NS | NS | case-control study | 51.2 | 8 | High | 170 | 142 |
| Chen YL 2004 ([14](#_ENREF_14)) | Shanghai | East China | Giemsa stain | Tissue | 2000-2002 | Before treatment | cross-sectional study | NS | 4 | Moderate | 86 | 63 |
| Xu XF 2004 ([15](#_ENREF_15)) | Fujian | East China | ELISA | Blood | 2001 | NS | case-control study | 79.0 | 7 | High | 105 | 30 |
| Li C 2005 ([16](#_ENREF_16)) | Hubei | South Central China | ELISA | Blood | NS | NS | case-control study | 66.1 | 7 | High | 59 | 55 |
| Huang YF 2005 ([17](#_ENREF_17)) | Fujian | East China | Rapid urease test or 13C urea breath test or IgG antibody detection | Tissue or breath or blood | 2003-2004 | NS | cross-sectional study | 81.3 | 4 | Moderate | 108 | 61 |
| Zhao DG 2006 ([18](#_ENREF_18)) | Henan | South Central China | Rapid urease test or pathological staining | Tissue | 2003-2006 | NS | cross-sectional study | 58.6 | 4 | Moderate | 102 | 82 |
| Liao SY 2006 ([19](#_ENREF_19)) | Guangdong | South Central China | ELISA | Blood | NS | Before treatment | case-control study | 60.6 | 9 | High | 104 | 85 |
| Song JQ 2007 ([20](#_ENREF_20)) | Hubei | South Central China | ELISA | Blood | NS | NS | case-control study | 58.7 | 8 | High | 143 | 74 |
| Dang JS 2007 ([21](#_ENREF_21)) | Shaanxi | Northwest China | 14C urea breath test | Breath | 1998-2003 | NS | cross-sectional study | NS | 4 | Moderate | 27 | 25 |
| Kamangar F 2007 ([22](#_ENREF_22)) | Henan | South Central China | ELISA | Blood | 1985-2001 | NS | cohort study | 66.5 | 8 | High | 343 | 276 |
| Deng WY 2008 ([23](#_ENREF_23)) | Fujian | East China | Rapid urease test | Tissue | 2004-2006 | NS | cross-sectional study | 80.0 | 3 | Low | 125 | 81 |
| Qiu HB 2009 ([24](#_ENREF_24)) | Guangdong | South Central China | PCR method | Tissue | 2002-2006 | Before treatment | cross-sectional study | 68.2 | 4 | Moderate | 108 | 51 |
| Wu IC 2009 ([25](#_ENREF_25)) | Taiwan | East China | Immunochromatographic screening test | Blood | 2000-2007 | Before treatment | case-control study | 62.8 | 8 | High | 167 | 119 |
| Jia A 2009 ([26](#_ENREF_26)) | Shaanxi | Northwest China | Rapid urease test or 14C urea breath test or IgG antibody detection | Tissue or breath or blood | 2005-2006 | NS | case-control study | 70.8 | 7 | High | 106 | 64 |
| Yu YH 2010 ([27](#_ENREF_27)) | Guangdong | South Central China | Rapid urease test or 13C urea breath test or 14C urea breath test | Tissue or breath | NS | NS | case-control study | NS | 5 | Moderate | 62 | 37 |
| Hu HB 2010 ([28](#_ENREF_28)) | Guangxi | South Central China | PCR method | Tissue | 2007-2008 | Before treatment | case-control study | 56.3 | 6 | Moderate | 238 | 142 |
| Zhang B 2012 ([29](#_ENREF_29)) | Inner Mongolia | North China | ELISA | Blood | 2008-2010 | NS | case-control study | 77.1 | 7 | High | 93 | 25 |
| Tao W 2012 ([30](#_ENREF_30)) | Ningxia | Northwest China | Giemsa stain | Tissue | 2010-2011 | NS | case-control study | NS | 6 | Moderate | 99 | 83 |
| Wang ML 2012 ([31](#_ENREF_31)) | Ningxia | Northwest China | Western blotting kit | Blood | 2009-2010 | NS | cross-sectional study | 57.1 | 3 | Low | 112 | 105 |
| Liang H 2012 ([32](#_ENREF_32)) | Henan | South Central China | ELISA | Blood | 1999-2007 | NS | case-control study | 53.5 | 8 | High | 127 | 97 |
| Guo BW 2012 ([33](#_ENREF_33)) | Hebei | North China | PCR method | Tissue | 2009-2011 | NS | cross-sectional study | 51.7 | 4 | Moderate | 130 | 96 |
| Wang DZ 2012 ([34](#_ENREF_34)) | Hebei | North China | Immunohistochemical staining | Tissue | 2005-2008 | NS | cross-sectional study | 74.7 | 4 | Moderate | 46 | 30 |
| Cao XY 2014 ([35](#_ENREF_35)) | Jilin | Northeast China | ELISA | Blood | 2000-2010 | NS | cross-sectional study | 76.2 | 8 | High | 103 | 67 |
| Zhang SS 2014 ([36](#_ENREF_36)) | Hubei | South Central China | Immunohistochemical staining | Tissue | 2011-2013 | NS | cross-sectional study | NS | 6 | Moderate | 19 | 9 |
| Long FR 2014 ([37](#_ENREF_37)) | Gansu | Northwest China | 14C urea breath test | Breath | 2012-2013 | Before treatment | case-control study | 54.4 | 8 | High | 86 | 63 |
| Hou YL 2014 ([38](#_ENREF_38)) | NS | NS | Warthin-Starry method or urease test | Tissue | NS | Before treatment | cross-sectional study | 53.3 | 3 | Low | 45 | 14 |
| Jiang LF 2014 ([39](#_ENREF_39)) | Jiangsu | East China | Rapid urease test or 13C urea breath test | Tissue and breath | 2012-2014 | NS | cross-sectional study | 64.0 | 4 | Moderate | 38 | 28 |
| Wang XQ 2014 ([40](#_ENREF_40)) | Shaanxi | Northwest China | ELISA | Blood | 2008-2010 | NS | case-control study | 69.0 | 8 | High | 171 | 101 |
| Zhang JX 2014 ([41](#_ENREF_41)) | Henan | South Central China | ELISA | Blood | NS | NS | case-control study | 75.4 | 8 | High | 65 | 46 |
| Wu YL 2015 ([42](#_ENREF_42)) | Henan | South Central China | 14C urea breath test | Breath | 2013 | Before treatment | cross-sectional study | 78.0 | 6 | Moderate | 128 | 85 |
| Kang L 2015 ([43](#_ENREF_43)) | Inner Mongolia | North China | ELISA or 14C urea breath test | Tissue or breath | 2011-2014 | Before treatment | cross-sectional study | 80.0 | 5 | Moderate | 138 | 87 |
| Zhou F 2015 ([44](#_ENREF_44)) | Jiangsu | East China | Immunohistochemical staining | Tissue | 2008-2013 | NS | cross-sectional study | 75.8 | 4 | Moderate | 100 | 76 |
| Guo BZ 2016 ([45](#_ENREF_45)) | Henan | South Central China | Ag-NOR argentophilic staining method | Tissue | 2014 | Before treatment | cross-sectional study | 56.7 | 3 | Low | 83 | 55 |
| Zhao J 2017 ([46](#_ENREF_46)) | Liaoning | Northeast China | Immunohistochemical staining | Tissue | 2014-2015 | Before treatment | cross-sectional study | 50.0 | 4 | Moderate | 85 | 63 |
| Gao AY 2017 ([47](#_ENREF_47)) | Heilongjiang | Northeast China | Immunohistochemical staining | Tissue | 2014-2017 | NS | cross-sectional study | 53.3 | 5 | Moderate | 10 | 9 |
| Fan CX 2017 ([48](#_ENREF_48)) | Gansu | Northwest China | 14C urea breath test | Breath | 2014-2017 | NS | cross-sectional study | 54.3 | 4 | Moderate | 37 | 31 |
| Wang RX 2017 ([49](#_ENREF_49)) | Shaanxi | Northwest China | rapid urease test or IgG antibody detection | Tissue or blood | NS | NS | case-control study | 70.8 | 9 | High | 106 | 64 |
| Yu M 2018 ([50](#_ENREF_50)) | Shaanxi | Northwest China | rapid urease test | Tissue | 2015-2016 | Before treatment | cross-sectional study | 53.3 | 4 | Moderate | 50 | 47 |
| Song Q 2019 ([51](#_ENREF_51)) | Inner Mongolia | North China | ELISA | Blood | 2016-2017 | Before treatment | cross-sectional study | 77.0 | 4 | Moderate | 87 | 40 |
| Lin J 2019 ([52](#_ENREF_52)) | Jiangsu | East China | IgG antibody detection | Blood | 2016-2017 | Before treatment | cross-sectional study | 63.6 | 7 | Moderate | 11 | 5 |
| Zhao YP 2020 ([53](#_ENREF_53)) | Jiangsu | East China | Methylene blue borate method | Tissue | 2019 | Before treatment | cross-sectional study | 52.0 | 5 | Moderate | 40 | 35 |
| Lin YL 2020 ([54](#_ENREF_54)) | Fujian | East China | Rapid urease test | Tissue | 2013-2015 | Before treatment | case-control study | 80.0 | 8 | High | 162 | 59 |
| Chen JY 2020 ([55](#_ENREF_55)) | Guangdong | South Central China | Immunohistochemical staining | Tissue | NS | NS | cross-sectional study | NS | 4 | Moderate | 95 | 63 |

CI, confidence interval; NS, not specific; PCR, polymerase chain reaction; ELISA, enzyme linked immunosorbent assay; IgG, [immunglobulin G](https://flexikon.doccheck.com/de/Immunglobulin_G); CLO, campylobacter-like organism; NOR, nucleolar organizer regions; *H. pylori*, *Helicobacter pylori*.

**Reference**

1. Webb PM, Yu MC, Forman D, Henderson BE, Newell DG, Yuan JM, et al. An apparent lack of association between *Helicobacter pylori* infection and risk of gastric cancer in China. *Int J Cancer* (1996) 67(5):603-7. Epub 1996/09/04. doi: 10.1002/(sici)1097-0215(19960904)67:5<603::aid-ijc2>3.0.co;2-y. PubMed PMID: 8782645.

2. Zhao C, Li Y. Correlation analysis between *Helicobacter pylori* and gastric disease. *Modern Journal of Integrated Traditional Chinese and Western Medicine* (1999) 8(9):1386-7.

3. Cai L, Yu SZ, Zhang ZF. The relationship between *Helicobacter pylori* infection and gastric cancer. *Cancer Research on Prevention and Treatment* (1999) 26(4):12-4.

4. Li ZY, Gao CM, Ding JH. Study on seroprevalence of *Helicobacter pylori* infection among upper digestive tract cancer patients and their kindreds. *Zhonghua liuxingbingxue zazhi* (1999) 20(2):88-90. Epub 2000/02/22. PubMed PMID: 10682540.

5. Lan CH, Leng ER. Clinical research on occurence of gastric cancer and infecyion of *Helicobacter pylori*. *Sichuan Medical Journal* (2000) 21(1):22-3.

6. Zhang XJ, Wu GL, Meng SQ, Ma JH. The relationship between *Helicobacter pylori* and 71 gastric a denocarcinoma cases. *Journal of Chinese Practical Medicine* (2000) 2(8):1-2.

7. Shen C, Bo S, Shao YW. Relation between chronic gastritis, non-cardia gastric cancer, and *Helicobacter pylori:* experience of a county hospital in China. *The American Journal of Gastroenterology* (2000) 95(9):2466.

8. Zhao DG. The relationship between *Helicobacter pylori* infection and tumor location in patients with gastric cancer. *Henan Journal of Oncology* (2001) 14(2):139-40.

9. Fei SJ, Chen YL, Liu ZF, Chen SM, Liu GZ, Li FC, et al. Relationship between *Helicobacter pylori* infection and expression of ras p2l and p53 in gastric carcinoma and preeancerosis. *Journal of Xuzhou Medical University* (2001) 21(4):288-91.

10. Zhang JQ, Li MM, Sun DA, Mao XY, Ding JY. Relationship between gastric cancer and *Helicobacter pylori* infection. *Chinese Journal of Clinical Gastroenterology* (2002) 14(5):214-5.

11. Hou YX, Wang QY, wang JY, Tian D. Relationship of *Helicobacter pylori* infection with location and type of gastric cancer. *Journal of Xi'an Jiaotong University(Medical Sciences)* (2003) 24(6):629-30+33.

12. Li YM, Zhao ZZ, li CZ, Tao J. The *Helicobacter PyIori* infection rate analysis of 85 earlierperiod cancer of stomach. *Journal of Chinese Clinical Medicine* (2003) 4(21):34-5.

13. Zeng ZR, Hu PJ, Hu S, Pang RP, Chen MH, Ng M, et al. Association of interleukin 1B gene polymorphism and gastric cancers in high and low prevalence regions in China. *Gut* (2003) 52(12):1684-9. Epub 2003/11/25. doi: 10.1136/gut.52.12.1684. PubMed PMID: 14633943; PubMed Central PMCID: PMCPmc1773879.

14. Chen YL, Zhang XP. The relationship between *Helicobacter pylori* infection and gastroduodenal diseases. *Medical Journal of Communications* (2004) 18(1):46-7.

15. Xu XF. Infection of CagA-positive *Helicobacter pylori* and the risk for cardia and non—cardia gastric cancer in high-risk area of China [Master]: Fujian University of Medicine (2004).

16. Li C, Xia B, Yang Y, Li J, Xia HX. Association of TNF gene polymorphism with *Helicobacter pylori* infection in Chinese patients with gastroduodenal diseases. *Chinese Journal of Immunology* (2005) 21(11):61-4.

17. Huang YF. The relationship among IL-1 gene polymorphism, *Helicobacter pylori* and gastric cancer development in Fujian Province [Master]: Fujian University of Medicine (2005).

18. Zhao DG, Pan JJ. The relationship between *Helicobacter* pylori infection and the positions of gastric cancer. *Public Medical Forum Magazine* (2006) 10(11):985-6.

19. Liao SY, Zeng ZR, Leung WK, Zhou SZ, Chen B, Sung JJ, et al. Peroxisome proliferator-activated receptor-gamma Pro12Ala polymorphism, *Helicobacter pylori* infection and non-cardia gastric carcinoma in Chinese. *Aliment Pharmacol Ther* (2006) 23(2):289-94. Epub 2006/01/06. doi: 10.1111/j.1365-2036.2006.02739.x. PubMed PMID: 16393309.

20. Song JQ, Xia B, Li C, Wu YH. Association between interleukin-1 gene polymorphisms and *helicobacter pylori* infection in non-cardiac gastric cancer in Chinese population. *Chinese Journal of Experimental Surgery* (2007) 24(4):451-3.

21. Dang JS, Chen WJ. Analyzing the related factors of *Helicobacter pylori* infection with gastric carcinoma of the ageol. *Hebei Medicine* (2007) 13(7):819-21.

22. Kamangar F, Qiao YL, Blaser MJ, Sun XD, Katki H, Fan JH, et al. *Helicobacter pylori* and oesophageal and gastric cancers in a prospective study in China. *British journal of cancer* (2007) 96(1):172-6. Epub 2006/12/21. doi: 10.1038/sj.bjc.6603517. PubMed PMID: 17179990; PubMed Central PMCID: PMCPmc2360212.

23. Deng WY, He LP, Peng XW. The relationship among IL-10 gene polymorphisms，*Helicobacter pylori* and noncardia gastric cancer development in Fujian province. *Chinese Journal of Gastroenterology and Hepatology* (2008) 17(3):212-4.

24. Qiu HB. Relationship between *Helicobacter pylori* infection and clinicopathological features and prognosis of gastric cancer [Master]: Sun Yat-sen University (2009).

25. Wu IC, Wu DC, Yu FJ, Wang JY, Kuo CH, Yang SF, et al. Association between *Helicobacter pylori* seropositivity and digestive tract cancers. *World J Gastroenterol* (2009) 15(43):5465-71. Epub 2009/11/17. doi: 10.3748/wjg.15.5465. PubMed PMID: 19916178; PubMed Central PMCID: PMCPmc2778104.

26. Jia A, Gong Y, Li YC, Chang XM, Hao ZM, Dong L. Association of IL-1β-511 and TNF-α-308 gene polymorphisms with non-cardia gastric cancer in Shaanxi Han population. *Journal of Xi'an Jiaotong University(Medical Sciences)* (2009) 30(1):70-3,127.

27. Yu YH, Chen CX, Huang QM, Zhang J. Association of Transforming Growth Factor Beta 1 Gene Polymorphism and Gastroauodenal Diseases with *Helicobacter pylori* Infection. *Chinese Journal of Clinical Gastroenterology* (2010) 22(5):273-6. doi: 10.3870/lcxh.j.issn.1005-541X.2010.05.05.

28. Hu HB, Liang XL, He JM. Interleukin 4 gene polymorphism, H? pylori infection and gastric cancer susceptibility in Liuzhou, Guangxi. *Journal of Guangxi Medical University* (2010) 27(1):48-51.

29. Zhang B. Roles of SNP rs4072037 in the MUC1 Gene in *Helicobacter pylori* Infection and Noncardia Gastric Cancer Risk in Baotou Han Population [Master]: Baotou Medical College (2012).

30. Tao W. Establish gastric cancer scoring models of high-risk population and Study the opportunistic screening method of Gastric cancer [Master]: Ningxia Medical University (2012).

31. Wang ML, Wang H, Yang L, Zhang YL, Wu YM, Li J. Study on association of *Helicobacter pylori* infection with gastric precancerous diseases and gastric cancer. *Ningxia Medical Journal* (2012) 34(2):114-6.

32. Liang H. Research on etiology and population attributable of environment risk factors of gastric and esophageal cancers [Doctor]: Peking Union Medical College (2012).

33. Guo BW, Yang L. Relationship between *Helicobacter pylori* genes cagE and virB11 and gastric cancer. *Chinese General Practice* (2012) 15(4C):1366-8.

34. Wang DZ, Zhang XH, Wu WX, Ma YM, Cui AR, Liu WN, et al. The relationship between the detection rate of *Helicobacter pylori* and Epstein-Barr virus latent membrane protein 1 in the tumor tissues of patients with cardia adenocarcinoma and distal gastric adenocarcinoma in Cixian County, Hebei. *Chinese Journal of Oncology* (2012) 34(6):446-9.

35. Cao XY, Cao DH, Jin MS, Jia ZF, Kong F, Ma HX, et al. CD44 but not CD24 expression is related to poor prognosis in non-cardia adenocarcinoma of the stomach. *BMC Gastroenterol* (2014) 14:157. Epub 2014/09/13. doi: 10.1186/1471-230x-14-157. PubMed PMID: 25212506; PubMed Central PMCID: PMCPmc4175630.

36. Zhang SS. Study on the correlation between the risk factors of *helicobacter pylori* infection and upper gastrointestinal disease in tumor high incidence [Master]: Hebei Medical University (2014).

37. Long FR. Pathological results and clinical analysis of *Helicobacter pylori* in patients with gastric cancer and gastric ulcer. *Guide of China Medicine* (2014) 12(31):178-9.

38. Hou YL, Wang GR, Qiu J, Sun SP. Relationship between *HP* infection and the expression of VEGF and MK in gastric cancinoma. *Journal of Modern Oncology* (2014) 22(10):2370-4.

39. Jiang LF, Wang Q, Niu QY, Zhao JB, Chen H. Analysis of Correlation between Pathologic Changes and *HP* Infection in Gastric Cancer. *Progress in Modern Biomedicine* (2014) 14(36):7087-9+129.

40. Wang XQ, Terry PD, Cheng L, Yan H, Wang JS, Wu WA, et al. Interactions between pork consumption, CagA status and IL-1B-31 genotypes in gastric cancer. *World journal of gastroenterology* (2014) 20(25):8151-7. Epub 2014/07/11. doi: 10.3748/wjg.v20.i25.8151. PubMed PMID: 25009387; PubMed Central PMCID: PMCPmc4081686.

41. Zhang JX, Duan GC, Guo AY, Chai J, Ma YH, Dong W. Study on the Relationship of IL-1B Gene Polymorphism and *Helicobacter Pylori* Infection with Gastric Cancer. *Medical Innovation of China* (2014) 11(27):1-3.

42. Wu YL, Cai Z, Wang WL. Relationship of *Helicobacter pylori* infection with mucosal inflammation in different types of gastric cancer. *Chinese Journal of Nosocomiology* (2015) 25(14):3192-3+240.

43. Kang L. Clinical study on the relationship between *Hp* infection and gastric cancer. *Inner Mongolia Medical Journal* (2015) 47(9):1115-7.

44. Zhou F. The sub-distribution of gastric cancer and the correlation between the expression of p53 and c-erbB-2 protein and *Hp* infection. *Journal of Clinical Medicine in Practice* (2015) 19(9):151-2.

45. Guo BZ. The relationship between *Helicobacter pylori* infection and the location and type of gastric cancer. *China Practical Medicine* (2016) 11(11):38-9.

46. Zhao J. Detection of *Helicobacter Pylori* in Patients with Gastric Cancer and Gastric Ulcer by Pathology and Clinical Analysis. *Guide of China Medicine* (2017) 15(8):1-2.

47. Gao AY. Detection of *Helicobacter Pylori* in Patients with Gastric Cancer and Gastric Ulcer by Pathology and Clinical Analysis. *China Health Care & Nutrition* (2017) 27(30):71-2. doi: 10.3969/j.issn.1004-7484.2017.30.099.

48. Fan CX. Analysis of Clinical and Pathological Results of *Helicobacter Pylori* in Patients with Gastric Cancer and Gastric Ulcer. *Doctor* (2017) 2(9):48+58.

49. Wang RX, Jia A. Correlation between gene polymorphisms of IL-10-819 and non-cardiac gastric cancer. *Chinese Journal of Medicine* (2017) 52(10):26-9.

50. Yu M, Wang B, Zhang YF, Tian YL, Liu JN, Li JH. Relationship between precancerous lesion and gastric cancer and *helicobacter pylori* infection. *Shaanxi Medical Journal* (2018) 47(11):1424-6.

51. Song Q, Gao F, Dong WJ, Liu DL, Ma LC, Wei XR, et al. Detection of *Helicobacter pylori* infection in Baotou Han population. *Journal of Baotou Medical College* (2019) 35(1):1-2.

52. Ji L, Liu ZC, Zhou B, Cai Y, An FM, Wang L, et al. Community-Based Pilot Study of a Screening Program for Gastric Cancer in a Chinese Population. *American Association for Cancer Research* (2019) 13(1):73-82. doi: 10.1158/1940-6207.capr-19-0372. PubMed PMID: 31796467.

53. Zhao YP, Yan G, Li M, Zhu RS. To study the results of pathological detection of *Helicobacter pylori* in patients with gastric cancer and gastric ulcer. *Electronic Journal of Clinical Medical Literature* (2020) 7(47):50+5.

54. Lin YL, Wu CC, Yan W, Guo SX, Liu BY. Five Serum Trace Elements Associated with Risk of Cardia and Noncardia Gastric Cancer in a Matched Case-Control Study. *Cancer Management and Research* (2020) 12:4441-51. doi: 10.2147/cmar.s250592. PubMed PMID: 32606938.

55. Chen JY, Xia D, Xu MM, Su RB, Lin WT, Guo D, et al. Expression and Significance of MyD88 in Patients With Gastric Cardia Cancer in a High-Incidence Area of China. *Front Oncol* (2020) 10:1-9. Epub 2020/06/02. doi: 10.3389/fonc.2020.00559. PubMed PMID: 32477927; PubMed Central PMCID: PMCPmc7239990.
